# New Osia® OSI200 active transcutaneous bone-anchored hearing device: how I do it

**DOI:** 10.1007/s00405-022-07786-w

**Published:** 2022-12-19

**Authors:** Dylan Chew, Vicki Proctor, Jaydip Ray

**Affiliations:** 1 Department of Otolaryngology, Sheffield Teaching Hospitals NHS Foundation Trust, Sheffield, UK; 2 Department of Hearing Services, Sheffield Teaching Hospitals NHS Foundation Trust, Sheffield, UK; 3 University of Sheffield, Sheffield, UK

**Keywords:** Bone conduction hearing, Mixed hearing loss, Auditory prosthesis

## Abstract

**Introduction:**

The new Osia® OSI200 implant incorporates a receiver coil and Piezo Power™ Transducer into one monolithic unit. Appropriate planning and surgical approach is needed for suitable positioning of the device.

**Method:**

To optimise the surgical field and provide tension-free wound closure our team have adopted a versatile ‘Sheffield-S’ post-auricular incision which remains hidden within the hairline.

**Conclusion:**

This incision provides adequate exposure for device placement and bone polishing/recessing. The soft tissue approach has resulted in improved operative efficacy particularly in those patients with irregular cortical bone or where pre-existing osseointegrated implants need to be removed or avoided.

## Introduction

The new Cochlear™ Osia® OSI200 System (Cochlear Ltd., Sydney, Australia) is an active transcutaneous bone-anchored hearing device with a piezoelectric actuator fixed to an osseointegrated titanium implant (BI300). The device is designed to aid hearing for those who have conductive, mixed or single-sided sensorineural hearing loss with pure tone average bone conduction threshold better than or equal to 55 dB (1). An external sound processor transmits power to the subcutaneous actuator via a digital radiofrequency (RF) link (1).

**Fig. 1:**
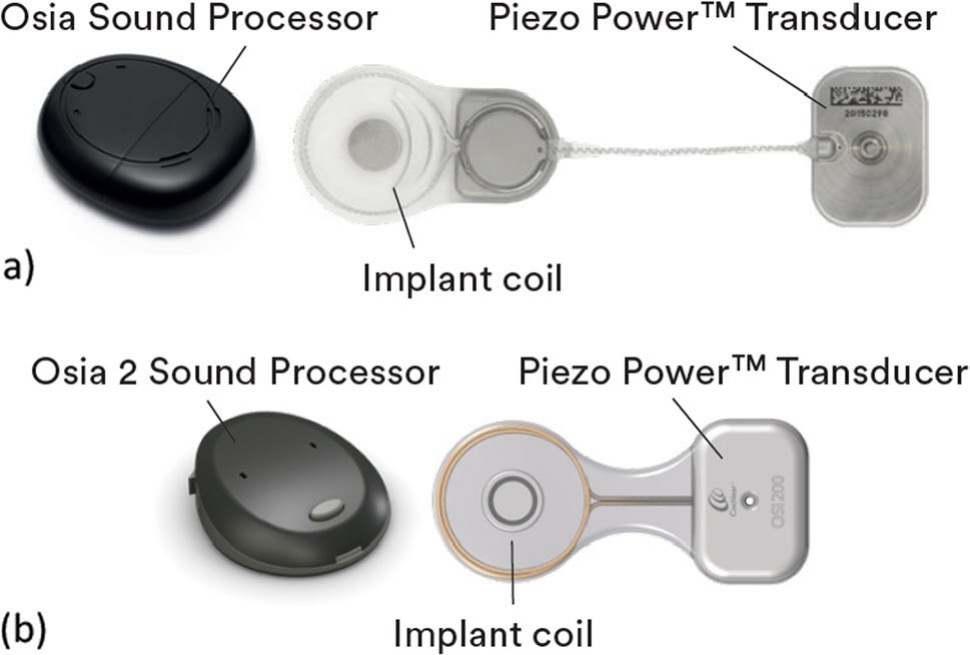
**a** First-generation Osia® system with OSI100 implant. **b** Second-generation Osia® system with OSI200 implant

The first generation Osia® system consisted of the OSI100 implant which had a flexible lead connecting the actuator with the coil/magnet module (Fig. 1a). The next generation OSI200 implant is ‘monolithic’ with an inflexible waist which maintains a fixed distance between the actuator and coil/magnet module (Fig. 1b). This design is intended to reduce feedback and simplify the surgical procedure. The external Osia® 2 sound processor is smaller than the previous model and has updated signal processing, wireless connectivity and more efficient power management (2).

**Fig. 2:**
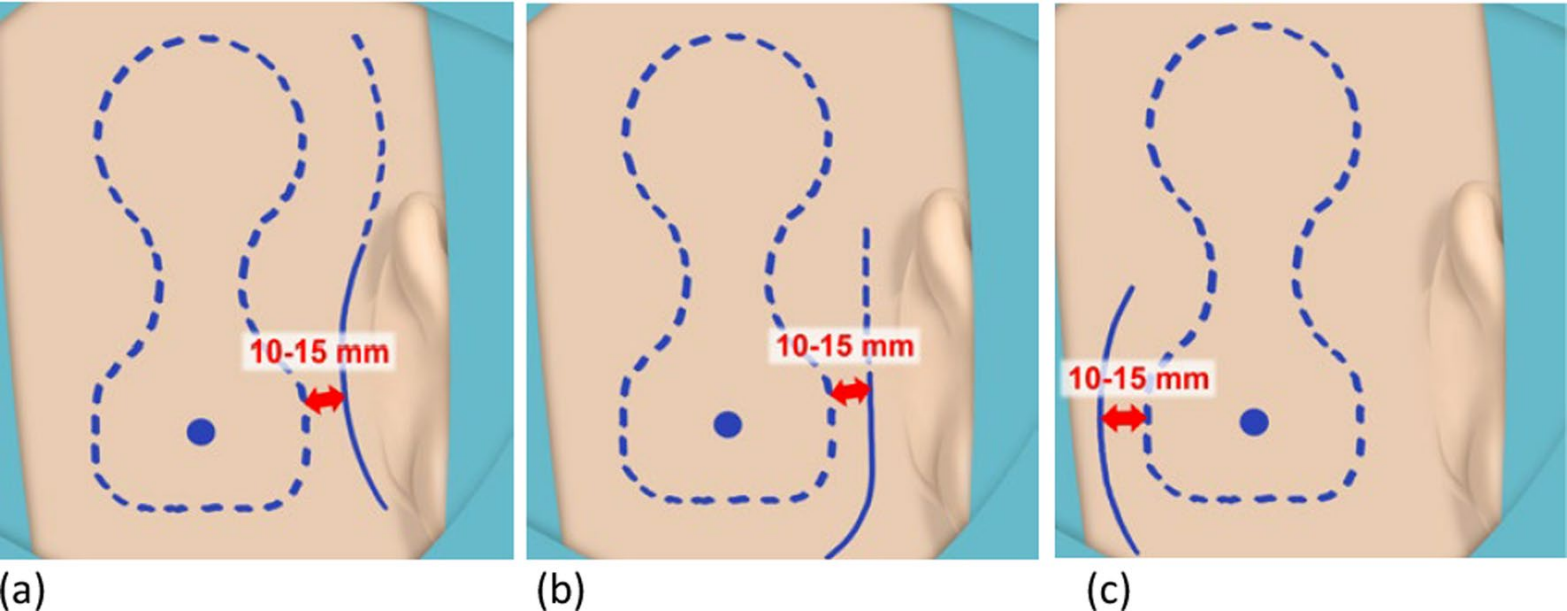
**a**–**c** Manufacturer recommended incisions for OSI200 implantation

The surgical guide provided by the manufacturer suggest the following surgical approaches: post-auricular incision, inferior post-auricular incision, posterior C-shaped incision (2) (Fig. 2a–c).

The recommendation to keep the incision 10–15 mm from the edge of the implant is to avoid tension on the skin and possible complications (2).

**Fig. 3:**
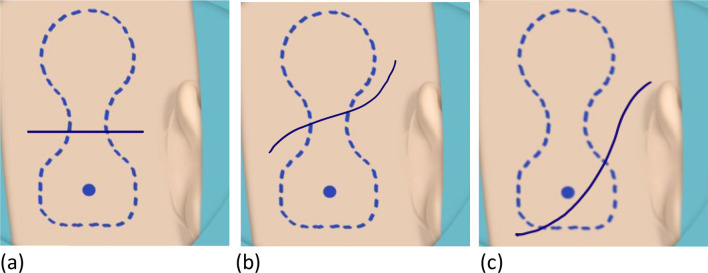
**a**–**c** Proposed alternative incisions for OSI200 implantation

At the time of writing this article, we have implanted 30 new OSI200 devices at our facility. We found the recommended incisions can be insufficient for an optimum surgical field in cases, where a pre-existing BAHA Attract® magnet needs to be removed; when a previously implanted titanium fixture needs to be identified in the bone for the new implant; or when the mastoid surface is irregular and bone polishing/recessing is required. Moreover, the incisions suggested by the manufacturer are adjacent to the free edge of the actuator which is the most prominent aspect of the device and more likely to encounter contact from objects worn behind the ear, such as glasses or face masks (increased usage following COVID-19 pandemic) (3, 4). By placing the incision across the waist (Fig. 3a, b) or surface of the actuator (Fig. 3c), a generous surgical field can be achieved allowing greater surgical efficacy and reduce surgical trauma. The curvilinear incision is similar to that used by neurosurgeons and neurotologists for the retrosigmoid approach to the internal auditory canal (IAC) and cerebellopontine angle (CPA) (5, 6).

## Methods and results

### Pre-operative planning

Physical examination of the patient is critical in the pre-operative assessment and planning to identify any previous scars and evaluate the skin quality and thickness behind the pinna. All our patients for Osia® implantation have a baseline CT scan of the temporal bones and an MRI scan to assess for aberrant anatomy and to exclude indolent intracranial pathology directly beneath the proposed implant site (as these may be obscured by metal artefact in future scans).

### Surgical technique

**Fig. 4:**
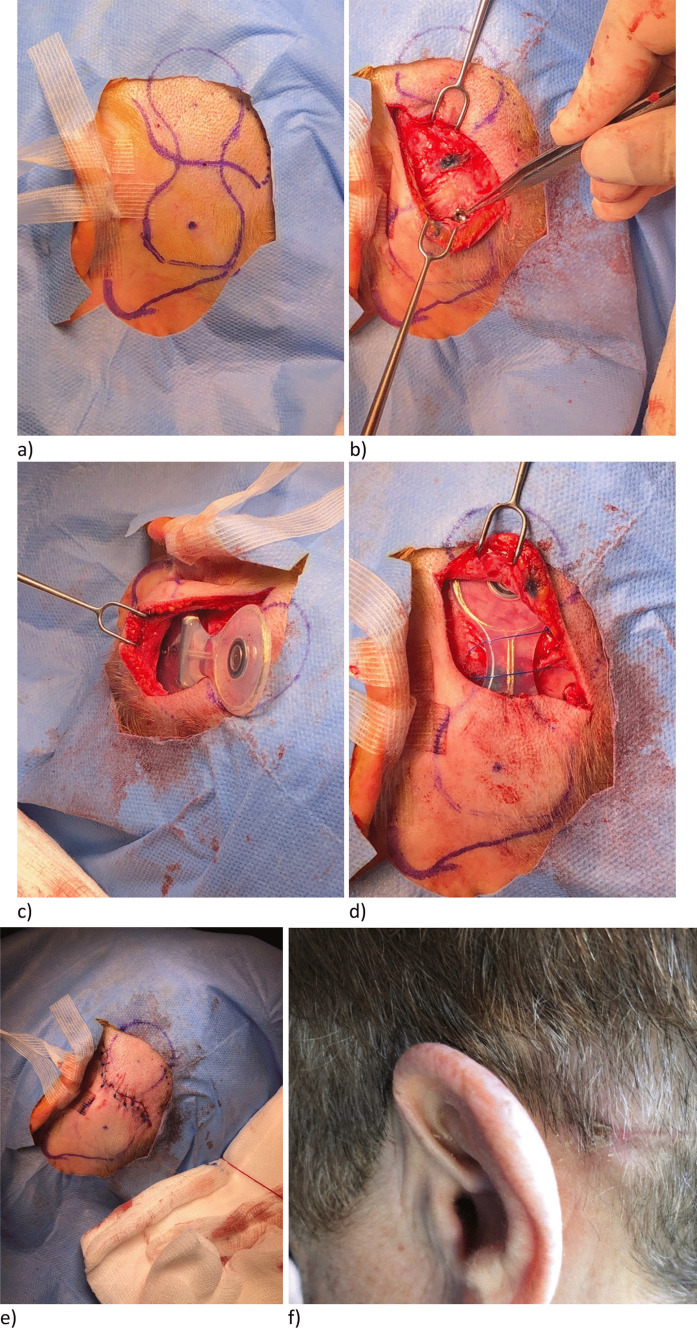
**a**–**f** Surgical steps for OSI200 implantation with curvilinear incision

Procedures have been performed under both general anaesthetic (28) and local anaesthetic (2). The patients’ average age was 53 years (range 24–79); there were 11 males and 19 females. 10 patients had devices implanted bilaterally and 20 had single-sided implantations. 21 patients had mixed hearing loss, while 4 had purely conductive hearing loss and 5 had sensorineural. The average skin thickness was 7.4 mm (range 5–8 mm); 4 patients (13%) required skin thinning and 16 patients (53%) required bone polishing/recessing. The patients are positioned supine with their head turned to the contralateral side and supported by a gel head-ring. A small arc of hair is shaved behind the pinna if required. Skin preparation and draping is as recommended by the manufacturer. The proposed position of the device is marked out using a skin marker and the OSI200 template provided by the manufacturer. The skin thickness over the proposed receiver–stimulator area is measured to plan for soft tissue thinning if required. The planned incision is then marked over the template site (Fig. 4a). Local anaesthetic with adrenaline is then infiltrated into the surgical site.

The incision is made down to but not incising the periosteum. Soft tissue pockets are created superiorly and inferiorly for device positioning. The template should be used again to confirm the position and mark the location for the titanium implant. At this stage the metal actuator template (if available) can be useful to evaluate the surface of the mastoid bone. Any instability of the template over the bone indicates possible need for bone polishing. Once the bone is satisfactorily smoothed the BI300 implant can be inserted as per the manufacturer’s instruction using the 3 mm or 4 mm guide drill to create the hole in the cortical bone, followed by the countersink drill and fixture placement (Fig. 4b). The bone-bed indicator can be used at this point to ensure adequate bony clearance for the actuator once docked with the BI300 implant. The OSI200 device can then be placed into the soft tissue pockets previously created and attached to the titanium implant with the fixation screw, with maximal torque of 25 Ncm (Fig. 4c, d).

Soft tissue is then closed in layers over the device—fascial layer and deep dermal layers with 3–0 undyed Vicryl and skin with 4–0 Ethilon (Fig. 4e). Non-adhesive dressings and a pressure bandage is then applied.

The resultant scar is well hidden within the hairline and we have not experienced any complications with skin tension or wound breakdown (Fig. 4f).

## Discussion

The new Osia® 2 device has had a positive reaction from our implanted patients thus far. They have been very pleased with the cosmesis, hearing function and impressed with the Bluetooth connectivity to auxiliary devices.

The surgical time has ranged from 30 to 90 min (average 40 min) depending on the complexity of the case. No surgical complications have been reported as yet. Our selection of incision based on pre-operative assessment has been free from any post-operative wound breakdown or skin complications. Other early experience of the Osia® device has shown a similarly low incidence of skin-related problems (7). Serious skin-related events have been reported in the literature, requiring the device to be explanted or replaced, but these were with the preceding OSI100 device (8, 9).

Initially we followed the manufacturers advice regarding skin incisions. For a native mastoid we found these incisions to be reasonable. However, after several cases we found these incisions could be insufficient for a number of reasons. First, if any bone polishing or recessing is necessary to correctly position the actuator over the mastoid with no tenting of the overlying skin (4), the recommended incisions often were not sufficient to expose the bone and keep soft tissues retracted to avoid trauma from the drill. In our experience, it is often not until the mastoid is inspected at the time of surgery that the need for polishing or recessing is clearly realised. Other published series have described the need for polishing/recessing of the mastoid cortex in the majority of cases (4, 9, 10). Another challenge is haemostasis, particularly controlling bleeding from emissary veins that may get damaged when elevating soft tissues. Without an adequate surgical field, one may be left blindly cauterising again leading to unnecessary soft tissue trauma or lead to post-operative haematoma (11).

Our preferred incision is now the curvilinear (Sheffield-S) across the waist of the implant. By choosing this incision primarily we avoid challenges with exposure if unforeseen difficulties present themselves during the surgery. The caveat to this approach is if previous post-auricular scars are present. In this instance we would routinely use the previous scar to avoid unsatisfactory cosmesis and potential skin complications.

## Conclusions

The ‘Sheffield-S’ incision described here has helped optimise the surgical field and provide a tension-free wound closure with the scar aesthetically well hidden within the hairline. This also provides adequate exposure for planning, placement and any bone polishing or recessing if required. There is improved operative efficacy in those patients with irregular cortical bone or since pre-existing osseointegrated implants need to be avoided.

## Key points

1. Curvilinear post-auricular incisions for implanting the Osia® OSI200 device offer the benefit of a wide surgical field with reduced skin tension when closing the wound.
2. Pre-operative planning with physical examination of the patient and imaging studies is essential for deciding which surgical incision will be most appropriate.
3. Following the incision and soft tissue dissection, create sufficient pockets for the actuator and coil/magnet with the aid of the device template.
4. Ensure your incision creates an adequate surgical field for any bone polishing/recessing required for optimum actuator placement. Adequate exposure of the mastoid bone will reduce the risk of unnecessary soft tissue trauma caused by drilling.
5. Once the BI300 implant has been screwed into the cortical bone, check that the wound can be closed over the actuator without wound tension or tenting of the overlying skin. Further undermine soft tissues if required.
6. Close the wound in layers.
7. Tension-free wound closure will reduce the risk of wound dehiscence and hair loss.

## Data Availability

The datasets generated during and/or analysed during the current study are available from the corresponding author on reasonable request.
